# Determinants of bacterial and fungal microbiota in Finnish home dust: Impact of environmental biodiversity, pets, and occupants

**DOI:** 10.3389/fmicb.2022.1011521

**Published:** 2022-11-07

**Authors:** Brandon Hickman, Pirkka V. Kirjavainen, Martin Täubel, Willem M. de Vos, Anne Salonen, Katri Korpela

**Affiliations:** ^1^Human Microbiome Research Program, Faculty of Medicine, University of Helsinki, Helsinki, Finland; ^2^Environmental Health Unit, Finnish Institute for Health and Welfare, Kuopio, Finland; ^3^Institute of Public Health and Clinical Nutrition, University of Eastern Finland, Kuopio, Finland

**Keywords:** bacteria, fungi, dust, indoor, environmental

## Abstract

The indoors is where many humans spend most of their time, and are strongly exposed to indoor microbiota, which may have multifaceted effects on health. Therefore, a comprehensive understanding of the determinants of indoor microbiota is necessary. We collected dust samples from 295 homes of families with young children in the Helsinki region of Finland and analyzed the bacterial and fungal composition based on the 16S rRNA and ITS DNA sequences. Microbial profiles were combined with extensive survey data on family structure, daily life, and physical characteristics of the home, as well as additional external environmental information, such as land use, and vegetational biodiversity near the home. Using permutational multivariate analysis of variance we explained 18% of the variation of the relative abundance between samples within bacterial composition, and 17% of the fungal composition with the explanatory variables. The fungal community was dominated by the phyla Basidiomycota, and Ascomycota; the bacterial phyla Proteobacteria, Firmicutes, Cyanobacteria, and Actinobacteria were dominant. The presence of dogs, multiple children, and firewood were significantly associated with both the fungal and bacterial composition. Additionally, fungal communities were associated with land use, biodiversity in the area, and the type of building, while bacterial communities were associated with the human inhabitants and cleaning practices. A distinction emerged between members of Ascomycota and Basidiomycota, Ascomycota being more abundant in homes with greater surrounding natural environment, and potential contact with the environment. The results suggest that the fungal composition is strongly dependent on the transport of outdoor environmental fungi into homes, while bacteria are largely derived from the inhabitants.

## Introduction

Humans may spend as much as 90% of their days indoors (Klepeis et al., 2001), and with the movement restrictions of 2020–2021 due to COVID–19, this proportion has likely increased drastically for many, in particular for the urban and suburban environments. The built environment represents a major source of microbial encounters, especially in the urban setting. Indoor environments harbor unique microbial ecosystems, which have been suggested to have a direct impact on human health (Berg et al., 2014; Adams et al., 2015; Martin et al., 2015; Shan et al., 2019). Exposure to a diverse microbial environment during early life has been often linked with protection against allergic diseases, in particular in the farming context (Ege et al., 2011). However, indoor microbes may also have a detrimental impact on human health, e.g., due to their immunogenicity or toxin production (Korkalainen et al., 2017), which is thought to be the mechanism contributing to the observed adverse health effects in moisture-damaged buildings (Mendell et al., 2011). Thus, not all indoor microbial diversity can be considered equally healthy, as for example showcased by the complex interrelations between moisture damage, fungal diversity, and asthma (Dannemiller et al., 2014).

The old friends hypothesis (Rook and Brunet, 2005) suggests that the microbes that humans coexisted with during our evolutionary history are beneficial and have a protective effect on our health. Such microbes may be scarce in the modern indoor environment. Indeed, several fungal species in the indoor microbiota have been linked with asthma (Dannemiller et al., 2014; Sharpe et al., 2014). One of the key features associated with directing immunological homeostasis early in life may be exposure to potential respiratory pathogens in comparison to microbes of the outdoor environment, e.g., soil origin (Kirjavainen et al., 2019). A key question is how to modify the indoor microbiota in a way that will promote beneficial microbial diversity without enriching harmful microbes.

A comprehensive understanding of the indoor microbiota and its modifiable determinants is essential to fully understanding how our modern human environment affects our health. The indoor microbiota has been shown to be affected by a range of variables relating to the specifics of the built environment itself, such as ventilation (Meadow et al., 2014; Barberan et al., 2015; Sharpe et al., 2020), and building type; the habits and actions of the individuals, such as the presence of pets (Fujimura et al., 2010; Kettleson et al., 2015; Mäki et al., 2021), cleaning habits (Bokulich et al., 2013), and occupancy (Täubel et al., 2009; Hospodsky et al., 2012; Cao et al., 2020); as well as the surrounding environment and geographic location (Leung and Lee, 2016; Weikl et al., 2016; Dockx et al., 2021). Farming, especially animal husbandry, has a strong influence on the indoor microbiota, as farm homes have very rich microbiota. Intriguingly not only is growing up on a farm protective of asthma but also growing up in homes, even urban homes, that have similar, farm home-like microbiota (Kirjavainen et al., 2019). Seasonal variation has been observed in fungal composition (Adams et al., 2013; Nevalainen et al., 2015) with little to no impact on bacterial composition (Rintala et al., 2008; Barberan et al., 2015). Well-ventilated homes have similar fungal microbiota composition as the outside environment and the hourly change of that composition mirror the outside over the course of a day (Meadow et al., 2014; Sharpe et al., 2020). The presence of pets has been shown to be associated with more diverse bacterial and fungal microbiota (Fujimura et al., 2010; Dunn et al., 2013; Mäki et al., 2021), and the number of occupants has been associated with increased abundance of human skin bacteria (Cao et al., 2020). A difference has been identified in the airborne microbiota composition between parks and parking lots (Mhuireach et al., 2016), and residential green spaces have been shown to have an effect on indoor bacterial and fungal diversity (Dockx et al., 2021).

While previous studies have mostly focused on specific variables, our objective was to obtain a comprehensive view of the determinants of indoor microbiota composition. Due to the high collinearity between many environmental variables, without careful adjustment for potentially confounding variables, the risk of identifying spurious correlations is high. We therefore collected a wide range of data to control for potential confounding effects. We examined the settled dust of 295 homes with young children (dust sampling was a part of the HELMi cohort (Health and Early Life Microbiota) (Korpela et al., 2019) which was designed to identify environmental, lifestyle, and genetic effects on infant intestinal microbiota) in the capital region of Finland and identified associations between indoor bacterial and fungal compositions, and a wide range of variables, such as the built environment, behavior and family structure, and the outdoor environment.

## Materials and methods

### Sampling and data

We utilized the Finnish birth cohort HELMi (Health and Early Life Microbiota) (Korpela et al., 2019). Settled dust samples (*n* = 295) were collected from households where at least one child in the household was between the ages 1.5 and 3 years old. The samples were collected from the room where the child spent the most time: living room (*n* = 186), kitchen (*n* = 9), combined living room and kitchen (*n* = 34), and other rooms (*n* = 66). Sampling was done in all seasons, but most samples were collected during winter (*n* = 189), while autumn (*n* = 32), spring (*n* = 15), and summer (*n* = 18) sampling was less common. The settled dust samples were collected using the Petri dish approach (Adams et al., 2015), exposing these passive dust collectors at a height of 120 to 220 cm with a minimum of 50 cm empty space above for a duration of 4 weeks. The sampling occurred between December 29^th^, 2018, and December 4^th^ 2020. No physical or chemical parameters of sampling location were collected, such as room temperature, relative humidity, CO2, or sampling room size, although housing type, apartment size, and room type were recorded. Petri dishes for dust sampling were handled with disposable vinyl gloves before and after sampling and closed with parafilm on site by study participants and posted to the analyzing laboratory, where they were stored for a maximum of 2 weeks at room temperature and in darkness until further processing.

The dust samples were accompanied by a survey collecting information regarding housing and building material, ventilation, number and type of pets, cleaning habits, and education. Additionally, we geolocated the homes and computed a host of environmental variables to accompany the survey data. The percentage of different land use classes were determined within 50, 250, 750, and 1,250 m buffers from each home utilizing the CORINE land cover 2018 (CLC, 2018) data (Copernicus Land Monitoring Service, European Union, 2018). Biodiversity zonation data describing the biodiversity of Finnish forests (Mikkonen et al., 2020) was also utilized to capture the biodiversity near the homes. Three biodiversity classes were utilized: NAT1, NAT2, and NAT6. NAT 1 is the lowest descriptive version giving only information on the degree of deadwood, exposing areas with lots of trees, tree species, and rare forest environments; NAT 2 adds penalties for forestry operations, taking human actions into account; NAT6 combines all biodiversity-related information and provides a list of the most valuable forest areas and landscapes. We utilized a total of 109 variables (S1) in our analysis.

### Dust sample processing and DNA extraction

Petri-dish dust sample processing and DNA extraction was performed as recently described in Dockx et al. (2021). In brief, four Petri dishes deployed in one home were thoroughly swabbed with a sterile cotton tip moistened in sterilized water +0.05% Tween 20, cotton tips were transferred into sterile, DNA LoBind Eppendorf tubes and stored at-80°C until DNA extraction. Blank samples consisting of four sterile, non-exposed Petri dishes were processed alongside the actual samples. At the time of DNA extraction, cotton tips were transferred into glass bead tubes containing 200 mg of glass beads, 400 μl lysis buffer of the Chemagic DNA Plant Kit (PerkinElmer chemagen Technology GmbH, Germany) were added, as well as salmon testis DNA (Sigma-Aldrich Co., United States) used as internal standard (Haugland et al., 2005). The suspension was subjected to mechanical cell disruption in a bead-milling step, using a MiniBeadbeater-16 (Biospec Products, Inc., United States) at maximum speed for 1 min. In the following steps, DNA was extracted and purified on a KingFisher™ mL DNA extraction robot (Thermo Fisher Scientific, Inc., Finland), following the manufacturer’s instructions for the Chemagic DNA Plant Kit protocol. Reagent and negative controls as well as bacterial and fungal mock communities were included in the DNA extraction process. DNA was stored at − 20°C and shipped on dry ice prior to amplicon sequencing.

One crucial aspect of the DNA extraction process, especially with respect to breakup of more rigid cells, is the bead beating step prior to DNA extraction and purification. The DNA extraction process method used includes bead beating step prior to DNA extraction and purification, building on the work earlier of Haugland et al. (2002) to improve extraction of a broad spectrum of microbes from environmental samples. Our analyses of samples include negative and positive controls, specifically mock communities of bacterial (6 strains) and fungal (30 plus strains) species, based on which the performance of DNA extraction can be monitored throughout the extraction process of a sample cohort. This DNA extraction approach has been successfully applied to several sample sets and matrices, including indoor dusts (Kirjavainen et al., 2019; Dockx et al., 2021), and air (Hyytiäinen et al., 2018) with downstream analyses being both quantitative (qPCR) and qualitative (sequencing) approaches.

### Dust microbiota analysis

Samples were prepared for parallel profiling of bacteria and fungi using 16S rRNA gene and internal transcribed spacer (ITS) region amplicons, respectively. The sequencing libraries were prepared as described by Virtanen et al. (2022) except for bacteria, where primers 515F (5’-GTGCCAGCMGCCGCGGTAA-3′) and 806R (5’-GGACTACHVGGGTWTCTAAT-3′) were used as described in Caporaso et al. (2011). The pooled 16S rRNA gene V4 and ITS amplicon mixture was sequenced at the Biomedicum Functional Genomics Unit (FuGU), Helsinki, Finland with an Illumina MiSeq instrument using paired-end 2 × 300 bp reads and a MiSeq v3 reagent kit. The loading concentration was 7.5 pM with 15% PhiX spike-in.

### Sequence processing

All samples were processed using the R package mare (Korpela, 2016). The reads were quality and chimera filtered, reverse and forward reads were merged using Usearch (Edgar, 2010). Amplicon sequence variants observed less than 138 times in the total dataset were excluded as likely errors. We used a minimum read relative abundance of 0.00001, assuming if rarer than this the result is likely an error. All amplicon sequence variants were assigned a taxonomy based on a Blast (Altschul et al., 1990) search against the NCBI prokaryotic 16S ribosomal RNA database and against the NCBI fungi ITS RefSeq Fungi database (Coordinators, 2016). Taxonomic tables were created based on the best hits. Of the fungal reads 58% (*N* = 223) were identified at species level (>95% sequence similarity to a reference sequence), 96.7% (*N* = 234) at genus level (>75% sequence similarity), and 83.3% (*N* = 5) at phylum level. From the bacterial data, 65.6% (*N* = 84) were identified at species level, 95.5% (*N* = 63) were identified at genus level, and 100% (*N* = 9) were identified at phylum level. A total of 1,963,179 bacterial and 4,458,027 fungal reads were annotated, with a mean of 6,655 and 15,111 per sample, respectively. Singletons were removed, and the data was not normalized nor rarefied.

### Statistical analysis

Statistical analysis was done using the R packages mare (Korpela, 2016) and vegan (Oksanen et al., 2016). Principal coordinates analysis (PcoA) was calculated with the capscale function of the R package vegan using Pearson correlation distances of the log-transformed data. To identify the most important variables determining microbiota composition, we performed permutational multivariate analysis of variance using distance matrices, with the Pearson distance, using the adonis function in the R package vegan to fit a linear model for both the bacterial and fungal data separately. All variables were combined, and the data tested against the bacterial taxa at species, genus, family, and order levels, as well as the fungal taxa at genus, family, order, and class levels. Fungal species data was not analyzed due to significant sparsity. We performed a stepwise model reduction, removing the variable with the largest value of p after each run. This continued until all remaining variables had a value of *p* <0.1. The identified final set of variables was tested for their association with individual microbial taxa.

Comparisons of the relative abundance of the bacterial and fungal genera were conducted using the GroupTest and CovariateTest functions in mare. The CovariateTest function was used with continuous data, while GroupTest was used for categorical data. These functions select an optimal model for each taxon using the lm function, glm.nb function from the MASS package (Venables et al., 2002) or the gls function from the nlme package (Pinheiro, 2021). The models were adjusted for the degree of ventilation, the type of pet, the number of times in the 3 months the sheets had been changed, the number of children living full time in the home, and the amount of arable land within 1,250 m. We chose confounders that represent the physical structure, the inhabitants, and the surrounding environment. The confounders were chosen from initial tests with the multivariate analysis models and were highly significant. The Standard Benjamini-Hochberg corrections for multiple testing were applied when testing multiple taxa to correct for false discovery rate (FDR) < 0.15, leading to the corrected value of ps <=0.04, but the uncorrected value of ps is reported for clarity in the Supplementary Table. Fold changes were calculated as the relative difference in the means between groups.

## Results

### Overall microbiota composition

**Figure 1:**
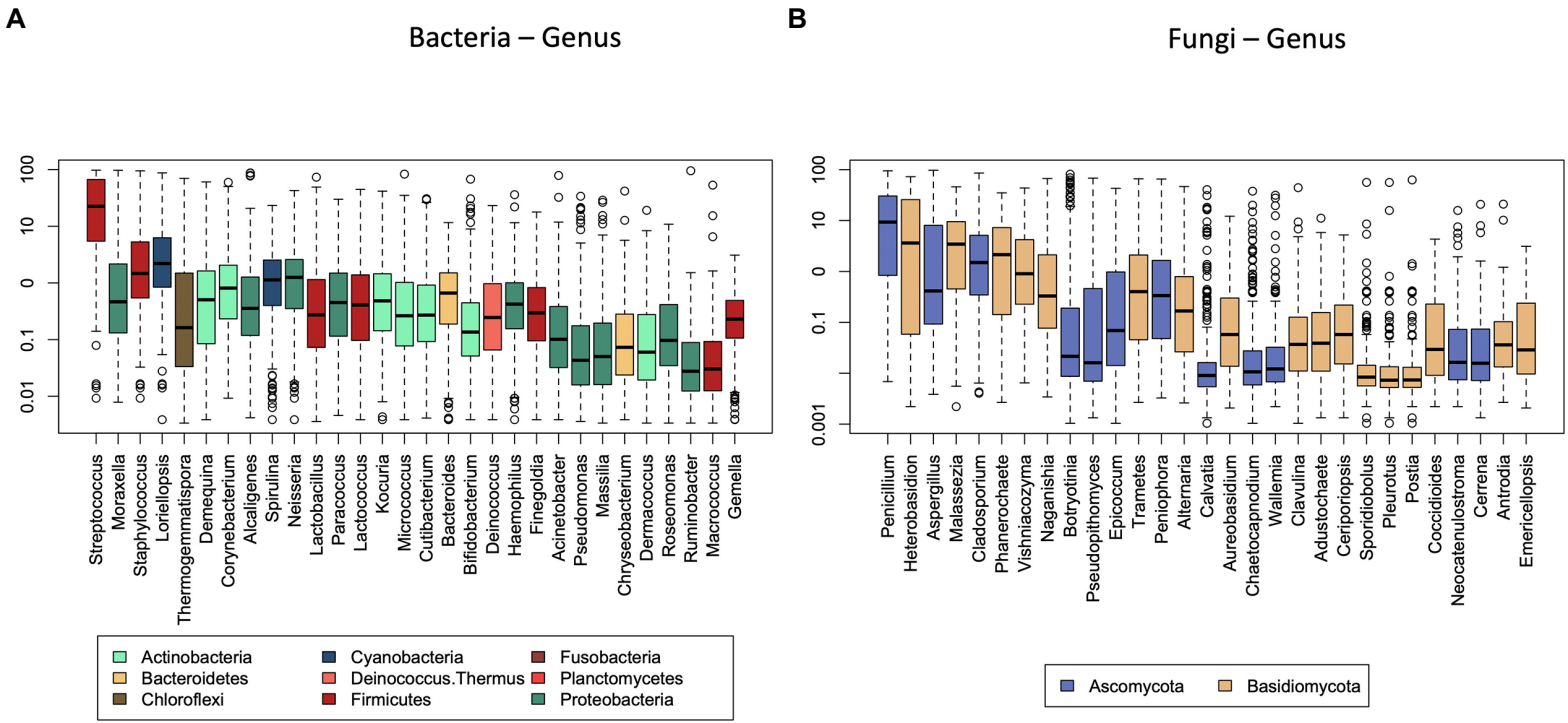
Median relative abundance of the thirty most abundant bacterial **(A)** and fungal **(B)** genera. Genera are colored by their corresponding phyla.

We identified 66 bacterial genera and 128 bacterial species in total in the different home dust samples. Thirty-three bacterial genera represented 90% of the total relative abundance of bacteria in the whole data set (Figure 1A). The composition at the genus level was highly dominated by *Streptococcus* (35%), followed by *Moraxella* (10%), *Staphylococcus* (6%), and the cyanobacterium genus *Loriellopsis* (6%). The remaining genera had a mean relative abundance below 5%.

The studied determinants explained 18% of the total variation in the relative abundance of different bacterial species between samples (Table 1). In the genus-level model, the significant determinants explained 9%, the family-level 11%, and the order-level 15%. All variables were also tested individually against the bacterial genera and reported with their *R*^2^ and *p*-values (S1).

**Table 1:** Multivariate analysis of variance results (*R*^2^ and value of *p*) for bacterial associations at the species-level.

Variable	*R* ^2^	*p*-Value
Firewood present	0.01051	0.053
House plants	0.00954	0.090
Building year	0.01170	0.026
Size of the apartment/house	0.01234	0.026
Building material	0.01784	0.082
Cold walls	0.01016	0.062
Father’s education	0.01034	0.046
Type of pet	0.02554	0.003
Regular use of baking soda, acetic acid, or another natural cleaner	0.01073	0.040
Number children living full-time in home	0.02076	0.013
Number children living part-time in home	0.01155	0.035
Transport 250 m	0.00956	0.081
Max biodiversity within 1,250 m (NAT1)	0.01091	0.038

In total, we identified 242 fungal genera and 383 fungal species. Fifty fungal genera represented 90% of the total relative abundance of fungi in the whole data set. The fungal biota were dominated by *Penicillium* (19%), *Heterobasidion* (15%), *Aspergillus* (10%), *Malassezia* (7%), *Cladosporium* (7%), and *Phanerochaete* (5%) with the mean relative abundance of the remaining genera below 5% (Figure 1B). For fungi, the significant determinants explained 17% of the compositional variation at the genus level, 16% of that at the order level, 14% at the family, and 16% at the class level (Table 2). All variables were also modeled using permutational multivariate analysis of variance using distance metrics individually against the fungal genera and reported with their *R*^2^ and *p*-values (S1).

**Table 2:** Fungal association results (*R*^2^ and *p*-value) for genus-level data (see Table 1).

Variable	*R* ^2^	*p*-Value
Firewood present	0.00828	0.032
Housing type	0.02468	0.001
Floor number	0.00983	0.008
Building year	0.01500	0.002
Fogging (windows)	0.00841	0.029
Number children living full-time in home	0.00747	0.042
Father’s education	0.01009	0.009
Type of pet	0.01794	0.001
Median biodiversity within 750 m (NAT1)	0.00761	0.038
Median biodiversity within 750 m (NAT2)	0.00735	0.051
Urban fabric 750 m	0.01345	0.002
Mine, dump, construction sites 750 m	0.00954	0.010
Arable land 1,250 m	0.00807	0.038
Pastures 1,250 m	0.01087	0.096
Season of collection: Winter	0.01126	0.005

### Pets

**Figure 2:**
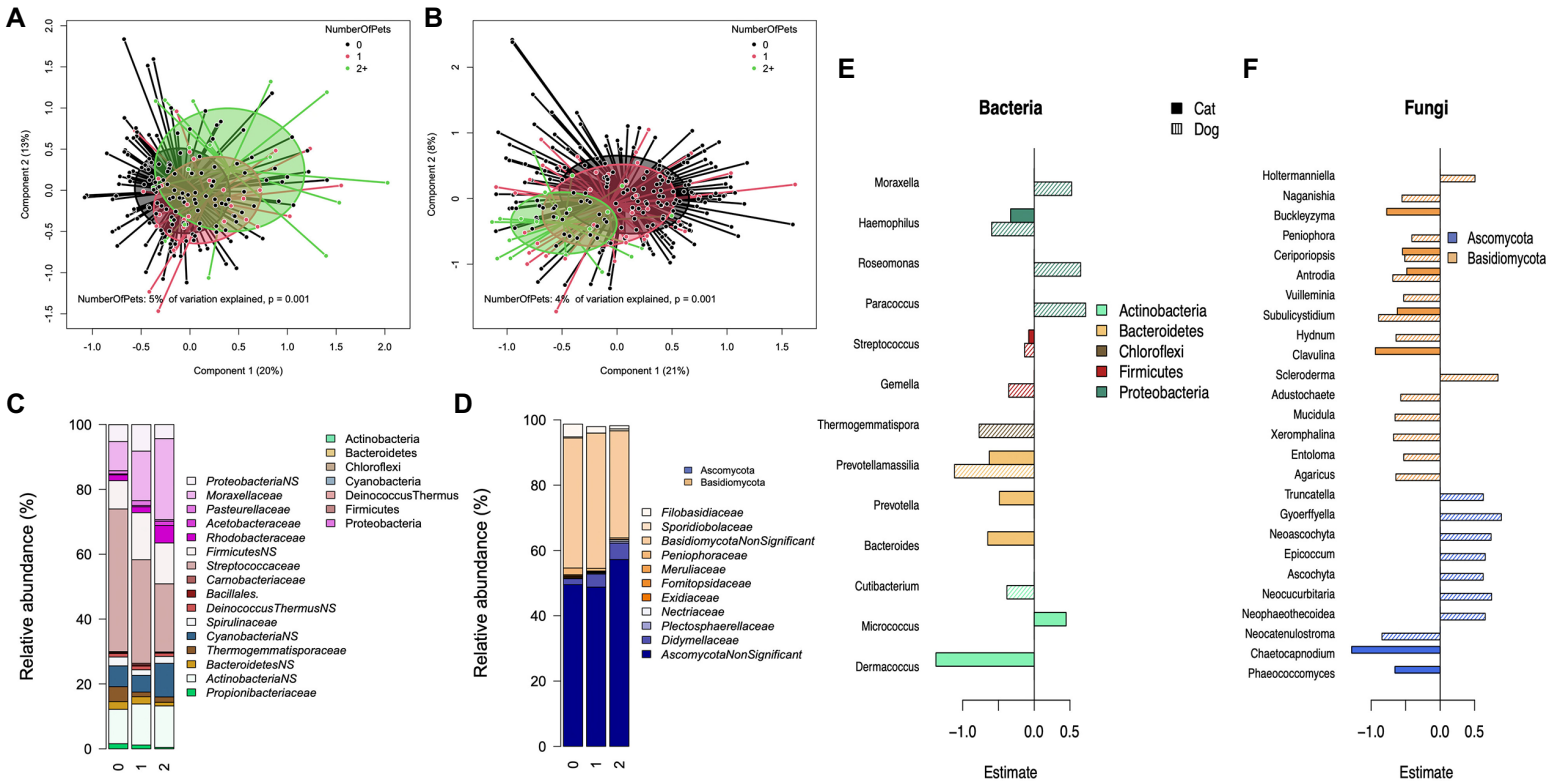
The role of the number of dogs in a household and bacterial and fungal composition. Principal coordinate analysis (PCoA) for the genus level composition of **(A)** bacteria and **(D)** fungi and grouped by the number of dogs in the home. Mean relative abundance of significantly and non-significantly (NS, *p*>0.07) associated bacterial **(B)**, and fungal **(E)** families by the number of dogs in the home. The estimated effect of the number of dogs, and cats on bacterial **(C)** and fungal **(F)** genera.

The type of pet in the home explained 4% (*p* = 0.001) of the variation in bacterial composition on the genus level in a permutational analysis of variance test. We performed stratified analysis on homes with either only dogs (*N* = 57) or no pets (*N* = 213) to assess the impact of the number of dogs (Figures 2A–C), and with homes with either no pets (*N* = 213) or only cats (*N* = 25) to assess the impact of the number of cats. There was a significant association between the number of dogs in the household and the first principal coordinate axis (*p* = 0.0003), with the number of dogs explaining 5% of the total variation (Figure 2A). In homes with cats or no pets (i.e., excluding dogs) the number of cats was significantly associated with the bacterial composition (*p* = 0.009), explaining 3% of the variation.

When excluding homes with cats, the number of dogs was inversely associated with several families within the phylum Firmicutes (*p*:0.013, Estimate: −0.103), particularly the family *Streptococcaceae* (*p*:0.001, Estimate: −0.139), *Prevotellamassilia* (*p*:0.009, Estimate: −1.12) within the phylum Bacteroidetes (Figures 2B,C). The number of dogs showed generally positive associations with families from the phylum Proteobacteria (*p*:0.002, estimate: 0.457), especially members of *Moraxellaceae* (*p*:0.036, estimate: 0.506) and *Rhodobacteraceae* (*p*:0.001, estimate: 0.720). When excluding homes with dogs, the number of cats was inversely associated with members of Bacteroidetes (*p* < 0.0001, estimate: 0.65) and positively with *Micrococcus* (*p* < 0.0001, estimate: 0.447) (Figure 2C). The presence of dogs in the home was also positively associated with the bacterial richness (*p* = 0.028, *R*^2^: 0.025).

Pet species explained 3% (*p* = 0.001) of the variation at the fungal genus-level relative abundance data. This appeared to be driven by the presence of dogs. When households with either dogs or cats were stratified, the number of dogs was significantly associated with fungal composition (explaining 4%, *p* = 0.0001, Figure 2D), but the number of cats was not (*p* = 0.224). The relationship between the number of dogs and fungal genera (Figures 2E,F) was divided between the phyla Ascomycota and Basidiomycota. There was a trend of increasing relative abundance of Ascomycota families, specifically *Didymellaceae* (*p* = 0.001; estimate: 0.59), with a corresponding decrease in Basidiomycota, such as *Filobasidiaceae* (*p* = 0.001; estimate: −0.60) and *Peniophoraceae* (*p* = 0.006; estimate: −0.52), with increasing number of dogs in the home (Figure 2E). The number of dogs was negatively associated with several genera from the phylum Basidiomycota, with an exception for the genera *Holtermanniella* (*p* = 0.002; estimate: 0.55) and *Scleroderma* (*p* = 0.005; estimate: 0.92), which showed a positive association with the number of dogs (Figure 2F). From the phylum Ascomycota, several genera had a positive association, with an exception for the genus *Neocatenulostroma* (*p* = 0.003; estimate: −0.88). The number of cats in the home showed a negative association with several genera irrespective of the phylum. The alpha diversity by pet type can be seen in Supplementary Figures 2A,B.

### Habitants and habits

**Figure 3:**
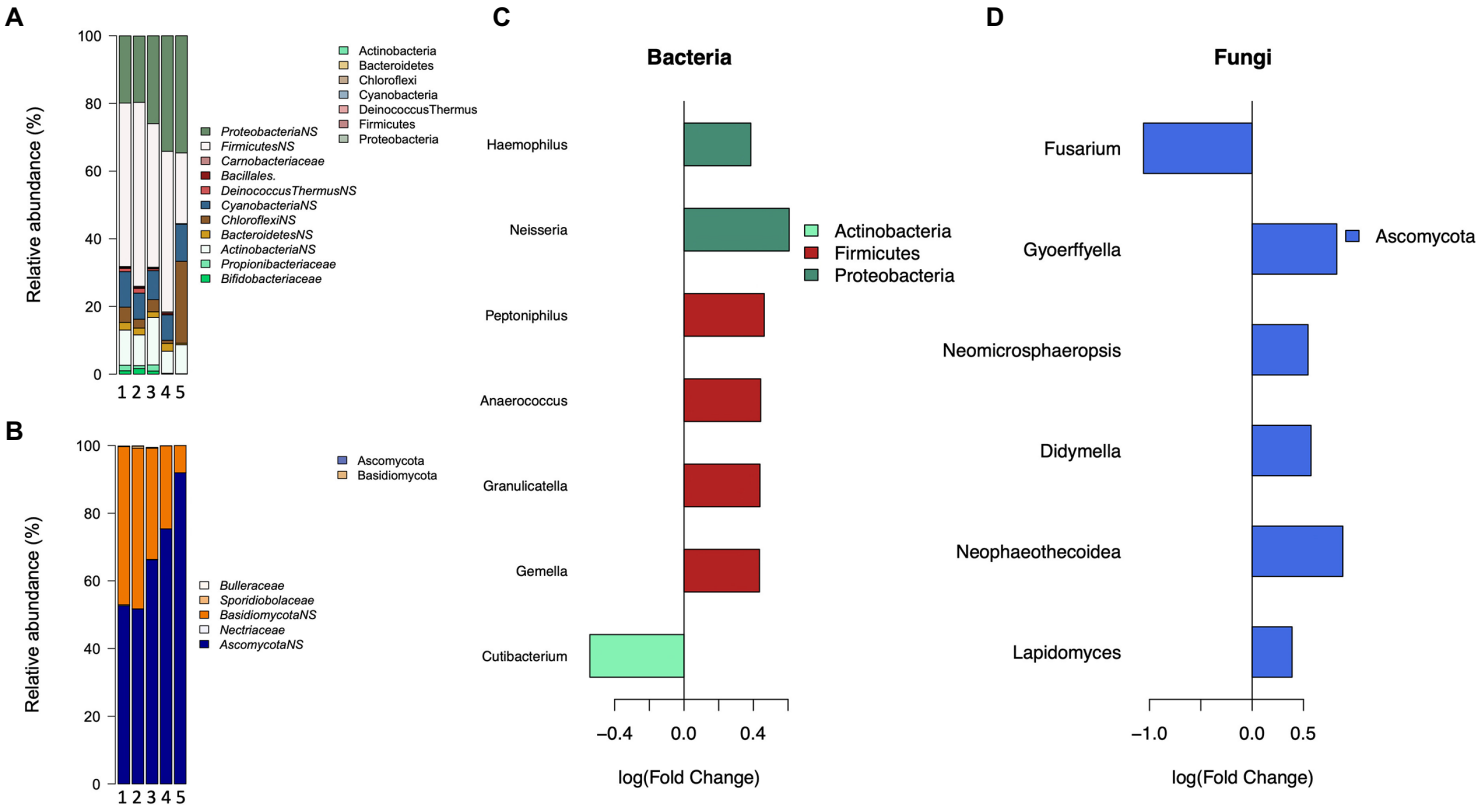
The association between the number of children in the home and bacterial and fungal communities. Mean relative abundance of significantly and non-significantly (NS, *p*>0.05) associated bacterial **(A)**, and fungal **(C)** families by the number of children in the home. The estimated effect of the presence of more than one child in the home on **(B)** bacterial and **(D)** fungal communities.

The number of children, both part-and full-time in the household had a significant association with the bacterial composition, with a trend of increasing relative abundance of Proteobacteria (Figure 3A). The presence of several children (2 or more) living full-time in the home (Figure 3B) had significant positive associations with members of Proteobacteria and Firmicutes, and a significant negative association with the genus *Cutibacterium* (*p* = 0.018, FC: −0.52) of the phylum Actinobacteria. The presence of children who only live part-time in the home had a significant negative association with genera *Dermacoccus* (*p* = 0.005, FC: 0.06), and *Moraxella* (*p* = 4.0e-05, FC: 0.29). For fungi (Figures 3C,D) a positive trend was observed for members of the Ascomycota phylum with the number of children living full time in the home, with several genera being significantly positively associated with the number of children. On the contrary, members of Basidiomycota had negative associations, with the family *Sporiodiobolaceae* (p:0.001; estimate: −0.44) significantly negatively associated with the number of children.

The father’s education had a significant positive association with the bacterial genus *Cutibacterium* (*p* = 0.002, estimate: 0.45), and negative associations with the genera *Staphylococcus* (*p* = 0.6e-03, estimate: −0.42), and *Acinetobacter* (*p* = 0.03, estimate: −0.35). Father’s education was also significantly associated with the fungal genera *Cladosporium* (*p* = 0.9e-03, estimate: −0.38) and *Crinipellis* (*p* = 0.001, estimate: −0.45). These results appeared to be driven by the six cases in the lowest education class. After removal of these cases, the associations were no longer significant. Neither the household’s highest education level nor the mother’s education level was associated with the bacterial nor fungal compositions.

The use of natural cleaning agents, such as baking soda or vinegar, was a significant variable in the bacterial model (*N* = 50, Table 1) and had a significant negative association with the genera *Cutibacterium* (*p* = 0.0005, estimate: −1.17), and *Paracoccus* (*p* = 0.0005, estimate: −0.98).

The presence of house plants had significant negative associations with the bacterial phylum Chloroflexi and genera *Thermogemmatispora* (*p* = 0.1e-5, FC = 0.714) and *Gemella* (*p* = 5.27e-03, FC = 0.62), and positive relationships were observed for the phylum Proteobacteria, in particular the genera *Massilia* (*p* = 0.002, estimate: 5.76) and *Moraxella* (*p* = 0.002, estimate: 2.657) (S2).

The number of times the bathroom was cleaned per month was positively associated with fungal richness (*p* = 0.019, *R*^2^ = 0.03). Firewood in the home explained about 2% (*p* = 0.002) of the variation in fungal composition at the genus level, and homes with firewood had a greater relative abundance of an unannotated family in the *Helotiales* class (*p* = 0.003, FC = 4.35) and lower relative abundance of the family *Extremaceae* (*p* = 0.002, FC = 0.26).

### Type of housing

**Figure 4:**
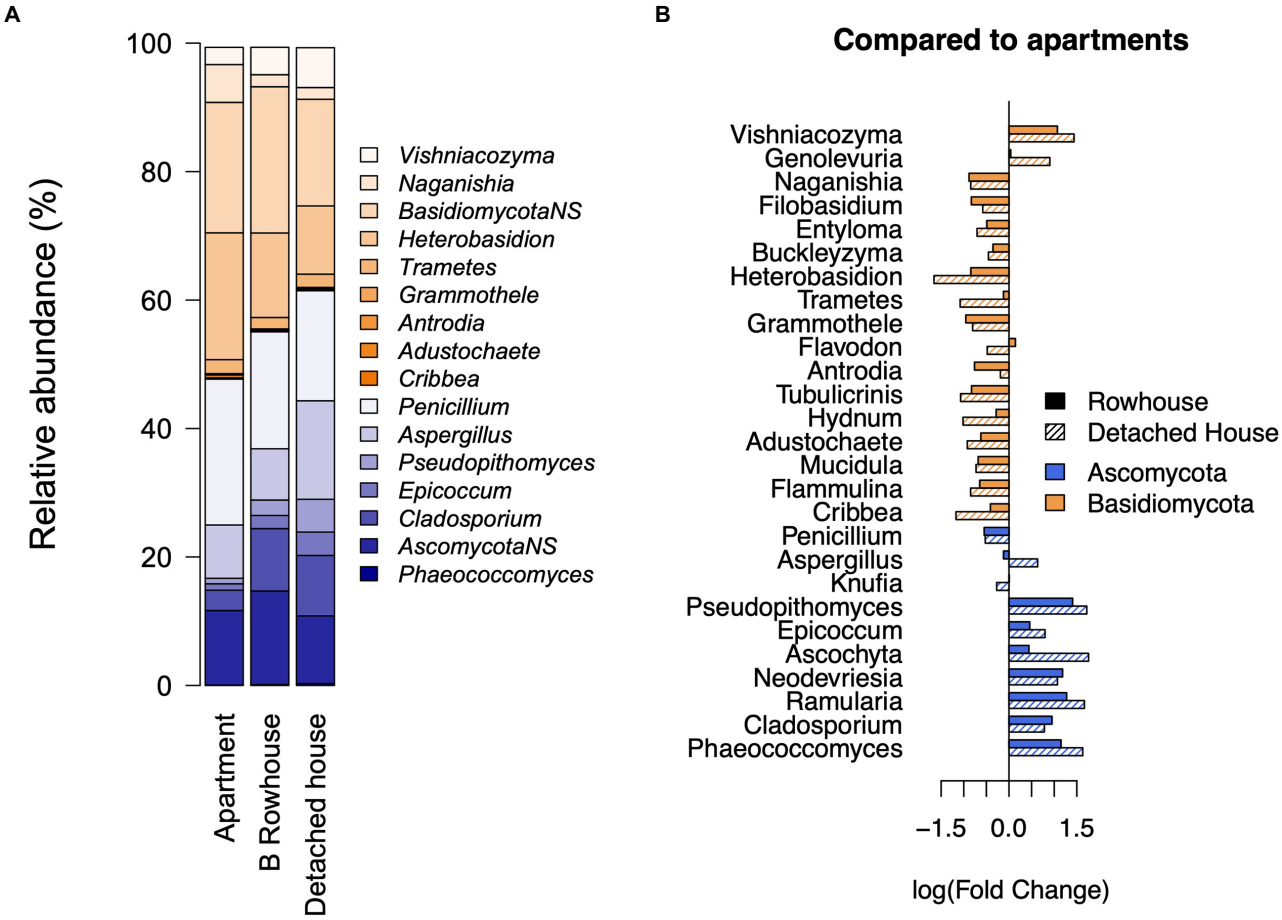
Role of the type of home on fungal community. **(A)** Mean relative abundance of significantly and non-significantly (NS, p>0.06) associated genera by type of home. Effect on genera when **(B)** compared to apartment buildings with rowhouses, and detached homes.

The building type explained 3% of the variation in fungal genus composition (*p* = 0.001), with a significant difference between detached and row houses from apartments (*p* = 9.917e-06). When compared to apartments (Figure 4B), a clear division between genera from the phyla Ascomycota (negative association) and Basidiomycota (positive association) emerged for both row and detached houses. Several Ascomycota genera, including *Aspergillus* (*p* = 0.03; FC: −0.63 from detached homes to apartment), *Pseudopithomyces* (*p* = 0.003; FC: −1.72), *Epicoccum* (*p* = 0.03; FC: −0.79), and *Cladosporium* (*p* = 0.0007; FC: −0.78), showed a significant association with building type, increasing in relative abundance from apartment buildings to rowhouses to detached homes (Figure 4A). Meanwhile, several genera from Basidiomycota, including the abundant genera *Naganishia* (*p* = 0.001; estimate: 0.84) and *Heterobasidion* (*p* = 0.003; estimate: 1.41), were associated with apartments (Figure 4B). The alpha diversity by house type can be seen in Supplementary Figures 2C,D.

The building material of the homes also explained 4% (*p* = 0.001). Building material was further investigated by stratifying by building type. All apartment buildings were constructed from stone, so detached houses and row houses were subdivided by building material. A significant difference (*p* = 0.004, variation explained = 4%) was observed between building materials for detached houses, but for rowhouses, there was no significant difference. There was a significant increase (*p* = 0.0007, FC = 3.52) in the relative abundance of the genus *Cladosporium* (Ascomycota), and a decrease in *Paracladophialophora* (Ascomycota; *p* = 0.006, FC = 0.23), and *Heterobasidion* (Basidiomycota; *p* = 0.004, FC = 0.08) in detached homes constructed from wood compared to those from stone/brick. No significant associations were found between building material and bacteria.

The floor number (apartment buildings only) from which the samples were collected had a significant association with fungal composition. The genera *Naganishia* (*p* = 0.001; estimate: 0.198), *Filobasidium* (*p* < 0.0001; estimate: 0.227), and *Buckleyzyma* (*p* = 0.002; estimate:0.126; Basidiomycota) were positively associated with increasing floor number, while *Ascochyta, Neodevriesia* (*p* < 0.0001; estimate: −0.399), *Ramularia* (*p* < 0.0001; estimate: −0.38), and *Phaeococcomyces* (*p* = 0.007; estimate: −0.196; Ascomycota) were more abundant in the lower floors (S2). There was a small negative association with building year for the bacterial genera *Paracoccus* (*p* < 0.0001; estimate: −0.38), and Acetobacteraceae (*p* = 0.0005; estimate: −0.013), while no association existed for fungal taxa and building year.

There was a significant negative association with the use of an air heat pump and bacterial richness (*p* = 0.035, *R*^2^: 0.022). However, no individual bacterial genus had a significant association with air heat-pump use. Floor heating in the room the sample was collected was inversely associated with bacterial richness (*p* = 0.039, *R*^2^: 0.023).

### Outdoor environment

**Figure 5:**
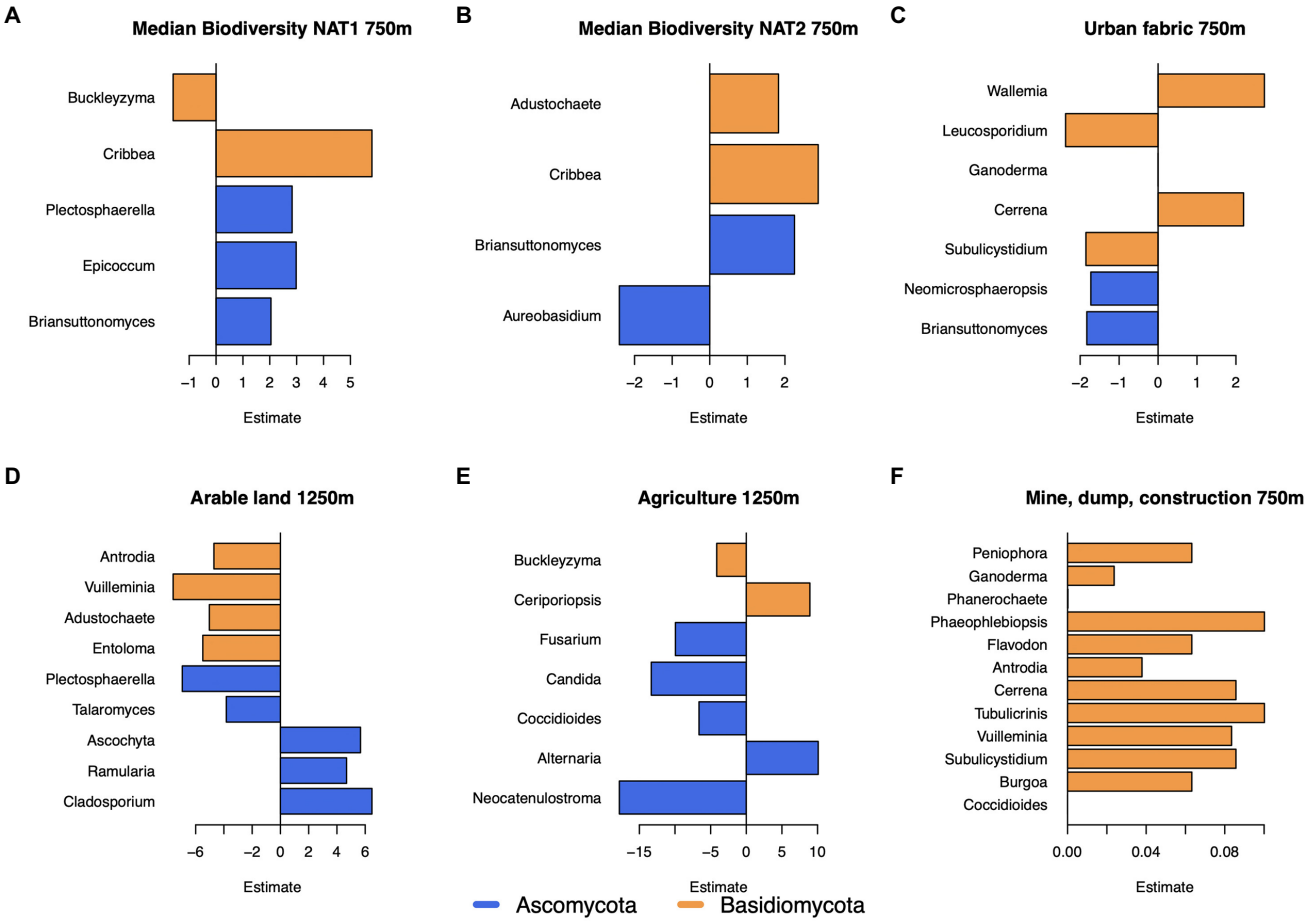
The estimated effect of the environement on fungal genera. **(A)** The median biodiversity (NAT1) within 750m, **(B)** the median biodiversity (NAT2) within 750m, **(C)** the degree of urban fabric within 750m, **(D)** the degree of arable land within 1250m, **(E)** the degree of agriculture within 1250m, and **(F)** the degree of mine, dump, or construction sites within 750m.

The land use classes urban fabric within 750 m; mine, dump, or construction sites within 750 m; arable land within 1,250 m, and pasture within 1,250 m were all associated with the fungal genus composition (Table 1). While the presence of agricultural land was not significant in the genus model, it was significant for all higher order models, thus it was also analyzed in detail. Increasing fraction of arable land was negatively associated with several Basidiomycota genera, while mine, dump, and construction land were positively associated with Basidiomycota genera (Figures 5D,F). Agricultural land was mostly negatively associated with Ascomycota genera (Figure 5E). While transport (250 m) remained in the bacteria model (Table 1), it was non-significant, and no significant associations with individual genera were found. The biodiversity variables – median biodiversity 750 m NAT1 and NAT2 – showed significant associations with several genera. The fungal genera *Cribbea* (NAT1: *p* < 0.0001, estimate:5.803; NAT2: *p* 0.009, estimate:2.885; phylum Basidiomycota) and *Briansuttonomyces* (*p* [NAT1] = 0.004, estimate:2.043; *p* [NAT2] = 0.002 estimate:2.554) (phylum Ascomycota) were positively associated with both biodiversity indicators (Figures 5A,B). The maximum value of biodiversity within 1,250 m (NAT1) was a significant variable in the bacterial model. Individually, the genus *Moraxella* (*p* = 4.55e-5, estimate = −0.46) and the genus *Roseomonas* (*p* = 0.002, estimate = −0.30) both had a small negative association with biodiversity.

The indoor dust fungal composition was significantly associated with the sampling season; however, most samples (75%) were collected during winter. Several genera from the phylum Basidiomycota showed a negative association with winter sampling, except for the genus *Cerrena* (*p* = 0.02, FC: 2.722) which had a positive association. The genus *Phaeococcomyces* (*p* < 0.0001, FC: 3.262) was the only significant genus from Ascomycota and it had a significant positive association with winter sampling (S2). Season of sampling was not significantly associated with the bacterial composition (*p* = 0.283). Relative abundance by season can be seen in Supplementary Figure 1 and the alpha diversity by house type can be seen in Supplementary Figures 2E,F.

## Discussion

We investigated the bacterial and fungal composition of indoor settled dust from homes with children aged 1.5–3 years old and the potential associations with the outdoor environment, occupancy, and occupant habits, and building characteristics. This study was conducted within the Helsinki capital area in Finland and represents mostly urban and suburban environments.

The bacterial composition in the indoor dust samples was dominated by 19 genera, from the Gram-positive phyla Actinobacteria, Chloroflexi, Cyanobacteria, Deinococcus, Firmicutes, and the Gram-negative phyla Proteobacteria, and Bacteroidetes. Previous studies (Bouillard et al., 2005; Rintala et al., 2008) have reported that homes are dominated by Gram-positive bacteria, which corresponds to our results, with Firmicutes alone representing 57% of the total abundance. Bouillard et al. (2005), and Rintala et al. (2008) have shown that Gram-negative taxa can represent about one-third of the bacteria in dust. Proteobacteria and Bacteroidetes, along with Firmicutes, were the most dominant phyla found in low-income housing (Ciaccio et al., 2015). In another study, Proteobacteria made up 22.4% of the bacteria in dust samples (Loo et al., 2018), which is comparable to the 24.6% relative abundance shown in our study. Indoor dust samples are typically dominated by human-associated bacteria genera: *Streptococcus*, *Staphylococcus, Corynebacterium,* and *Lactobacillus* (Hanson et al., 2016). While *Streptococcus* and *Staphylococcus* represented roughly 42% of the total abundance in our data, additional genera *Moraxella* (11%), *Loriellopsis* (7%), Corynebacterium (2.5%) and Lactobacillus (2%) were also present. Limitations in sampling settled dust can lead to bias in what microbes are collected. Settled dust leans more heavily towards larger particle sizes, as smaller bodied microbes may remain suspended in the air. This may lead to underrepresentation of smaller microbes in the sample population (Konya and Scott, 2014). The dishes remained in place for 4 weeks, increasing the likelihood of capturing also smaller microbes. Adams et al. (2015) showed that sampling at an elevated location in a home upon Petri dishes meets all the necessary criteria for collecting microbial community composition.

Hanson et al. (2016) reported that at the class level fungal microbiota in residential home dust was dominated by *Dothideomycetes*, *Agaricomycetes*, and *Tremellomycetes*. We observed the same dominant classes in our study, in addition to *Eurotiomycetes* (including the major filamentous fungal genera *Aspergillus* and *Penicillium*), and the likely human-associated yeast family *Malasseziomycetes*. The fungal composition was dominated by two phyla, Ascomycota and Basidiomycota, as has been observed in previous studies (Hanson et al., 2016; Gangneux et al., 2020). In our study, we saw on average a near equal divide between Basidiomycota and Ascomycota, while Hanson et al. (2016) reported an Ascomycota-dominated composition from settled dust (vacuumed). However, we found a clear division between the fungal phyla, with members of Ascomycota being associated with detached homes and low floors, the presence of dogs, several children, detached homes, and high biodiversity in the surrounding area. Conversely, members of Basidiomycota were negatively associated with dogs, relatively more abundant in apartments and higher floors and near dump, mine, or construction areas, and less abundant during winter. This indicates that members of Ascomycota tend to be relatively more abundant in homes with greater access to the natural environment, while members of Basidiomycota appear to dominate homes less access to the environment and in areas with lower biodiversity.

Ascomycota are important decomposers in soil ecosystems (Egidi et al., 2019), and they are likely transported into homes in the soles of shoes and feet of dogs. While wearing shoes inside one’s home in Finland is unusual, dogs in cities typically live inside. Basidiomycota, on the other hand, includes mushroom-type macrofungi that reproduce *via* spores, that spread through air, and that are more easily transported longer distances and even into higher-floor apartments. Snow cover during winter could explain the seasonality in the relative abundance of members of Basidiomycota. Indeed, indoor dust in detached homes has been described as dominated by Ascomycota, while outdoor ambient air particulates by Basidiomycota (Hanson et al., 2016). This dominance in outdoor ambient air could explain the increase in relative abundance of Basidiomycota in apartments that have low exposure to soil fungi. The compositional nature of the data makes it difficult to identify real effects on abundance, since if one dominant taxon increases in relative abundance, others will decrease correspondingly, even if they are not truly affected (Jian et al., 2020). It is likely that the differences in the relative abundances of Ascomycota and Basidiomycota are driven by changes in the abundance of Ascomycota. Members of soil-dwelling Ascomycota are likely more dependent on transportation by occupants into homes, and their abundance is thus affected by contact with the environment. Conversely, the air-borne spores of Basidiomycota likely are less affected by physical proximity and contact to the environment, and their abundance most likely is more equally distributed.

The type of building (e.g., hospital, schools, housing) has been linked to the bacterial community composition in a meta-analysis (Adams et al., 2015). Airborne bacterial and fungal concentrations and size distribution has been shown to vary between building types (Nasir and Colbeck, 2010). In our data, housing type and building material had a significant impact on fungal, but not bacterial composition.

In line with previous studies (Fujimura et al., 2010; Kettleson et al., 2015; Mäki et al., 2021), the presence of dogs was associated with both the fungal and bacterial compositions. Bacterial richness was significantly larger in homes with dogs than in homes without dogs. With increasing number of pets, a trend of increasing relative abundance was seen in the genera *Paracoccus* and *Roseomonas*, both commonly abundant in natural environments (Jiang et al., 2006; Czarnecki and Bartosik, 2019; Jeon et al., 2019) with a decrease in the relative abundance of the human-associated genera *Cutibacterium* (skin), *Prevotellamassilia, Streptococcus,* and *Gemella* (gastrointestinal). Several Proteobacteria genera were associated with dogs, corresponding to observations by Mäki et al. (2021). Indeed, Proteobacteria is a main phylum to inhabit both the skin and gut of canines (Pilla and Suchodolski, 2020; Mäki et al., 2021), while the relative abundance of Actinobacteria is higher in felines (Handl et al., 2011; Moon et al., 2018).

While the importance of dogs on the bacterial composition has been shown in previous studies (Dunn et al., 2013; Sitarik et al., 2018), dogs have been shown to have a lesser impact on the fungal compositions (Barberan et al., 2015; Kettleson et al., 2015). However, our findings show that the presence of dogs has an association with the fungal composition. While dogs appear important for indoor microbiota, the role of cats is modest. A likely explanation is that dogs spend time outdoors, transporting environmental microbes into the home, while city cats are largely indoor pets. The presence of multiple children was also associated with both bacterial and fungal compositions, with several human gut-associated bacteria of the Proteobacteria and Firmicutes phyla and environmental fungal taxa being positively associated with children.

The proportion of arable land, agriculture, and urban fabric, and proximity to construction or dump sites in the vicinity of the homes was associated with the fungal community composition. In addition, biodiversity indicators were significantly associated with both the fungal and bacterial communities. However, the great majority of the dust samples were collected during winter, when the ground was frozen and covered in snow, likely reducing the impact of the environment. Season (wintertime sampling) was associated with the fungal communities. Seasonality has been seen as an important constraint on both bacterial and fungal composition in indoor dust microbiota, as the natural outdoor sources are frozen (Weikl et al., 2016). Wintertime is the season when children are inside much of the day and thus represents the longest exposure season for indoor microbiota. Wintertime sampling could also account for the importance of firewood for both bacteria and fungi, as wood burning is typically only present in cold seasons, and therefore firewood would be absent from the home in warm seasons.

By testing a large number of determinants, we discovered that exposure to the environment was important for fungal composition, while inhabitants and their practices were important for bacterial composition. This likely reflects the different sources of indoor fungi and bacteria: fungi likely arise mostly from the environment, transported either in shoes or *via* air, while bacteria are mainly derived from the inhabitants. Identification of the indoor dust compositions with health: such as allergy, asthma, or overall wellbeing, would be an important next step. Additionally, any specific association with exposure to Basidiomycota or Ascomycota do their apparent inverse relationship in indoor dust shown in this paper.

## Data availability statement

The data presented in the study are deposited in the NCBI repository, accession number PRJNA892469.

## Author contributions

BH analyzed the data and wrote the manuscript. KK supervised the data analyses and contributed to writing the manuscript. WV and AS contributed to designing the study, revising the final draft, designing the study, and revising the final draft. MT and PK substantial contributions to interpretation of data and revisions of the drafted work. All authors contributed to the article and approved the submitted version.

## Funding

This work was supported by Emil Aaltonen Foundation (308254), Juho Vainio Foundation (1325103), and Academy of Finland (1308255).

## Conflict of interest

The authors declare that the research was conducted in the absence of any commercial or financial relationships that could be construed as a potential conflict of interest.

## Publisher’s note

All claims expressed in this article are solely those of the authors and do not necessarily represent those of their affiliated organizations, or those of the publisher, the editors and the reviewers. Any product that may be evaluated in this article, or claim that may be made by its manufacturer, is not guaranteed or endorsed by the publisher.

## Supplementary material

The Supplementary material for this article can be found online at: https://www.frontiersin.org/articles/10.3389/fmicb.2022.1011521/full#supplementary-material
