# Supplementary material for: Determinants of bacterial and fungal microbiota in Finnish home dust: Impact of environmental biodiversity, pets, and occupants
Source: Front Microbiol. 2022 Nov 7;13:1011521. doi: 10.3389/fmicb.2022.1011521 (PMC9676251; doi:10.3389/fmicb.2022.1011521)
Supplement: Supplementary file 3 [file Presentation_1.PPTX]

## Slide 1
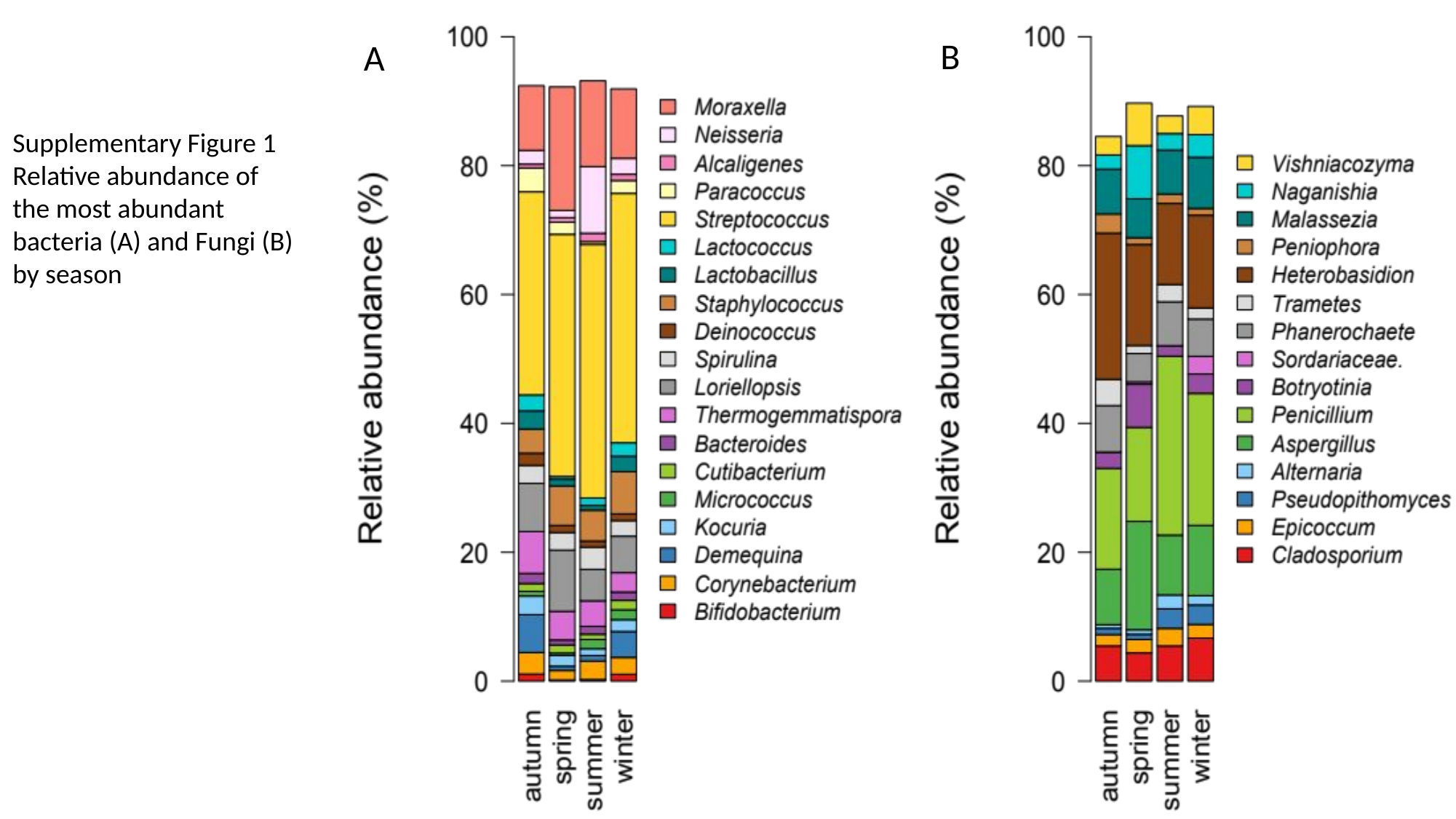

B
A
Supplementary Figure 1
Relative abundance of
the most abundant
bacteria (A) and Fungi (B)
by season

## Slide 2
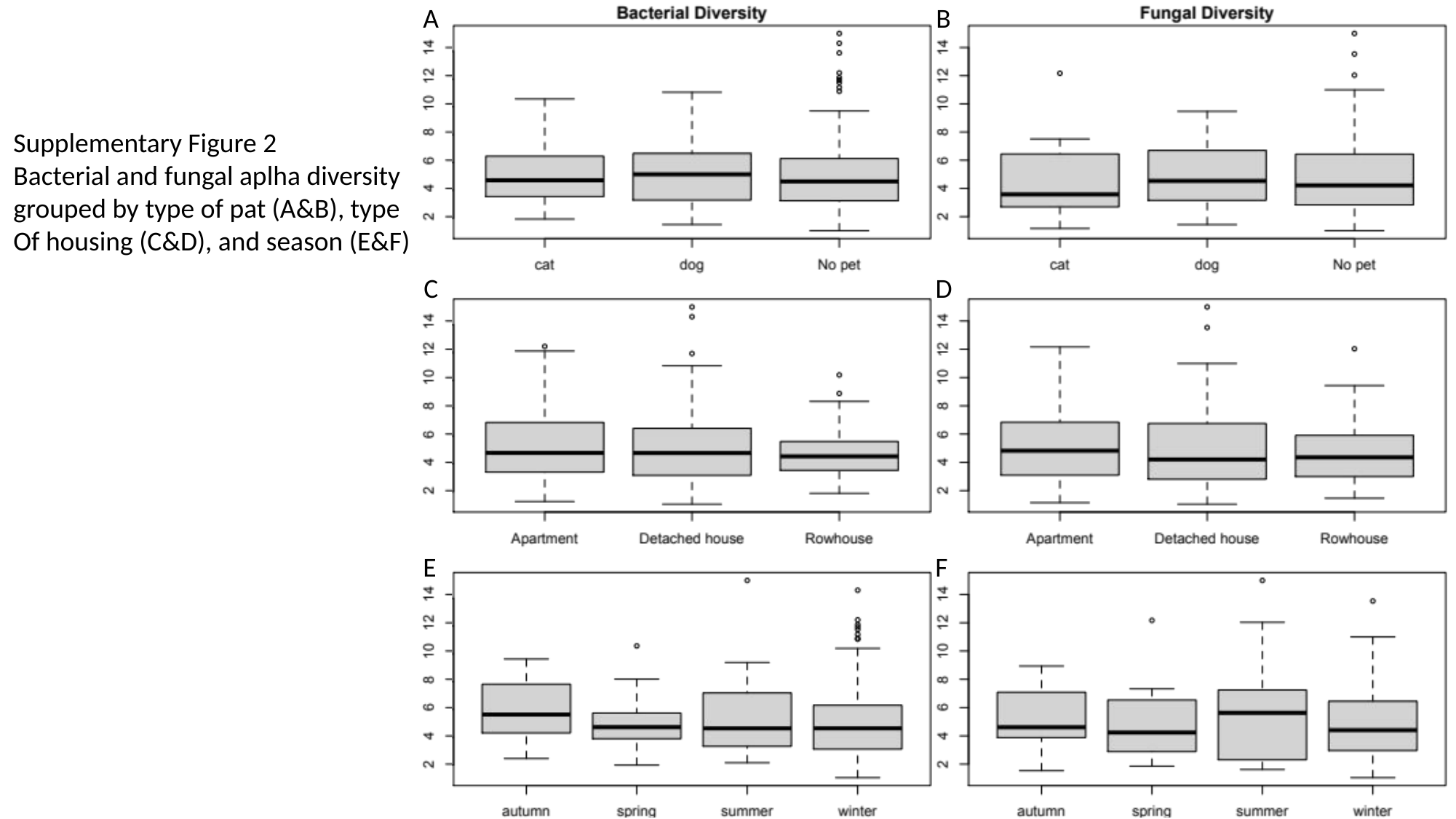

B
A
D
C
F
E
Supplementary Figure 2
Bacterial and fungal aplha diversity
grouped by type of pat (A&B), type
Of housing (C&D), and season (E&F)
